# The Influence of Arabinoxylans on the Properties of Wheat Bread Baked Using the Postponed Baking Method

**DOI:** 10.3390/molecules29040904

**Published:** 2024-02-18

**Authors:** Angelika Bieniek, Krzysztof Buksa

**Affiliations:** Department of Carbohydrate Technology and Cereal Processing, University of Agriculture in Krakow, Balicka 122, 30-149 Krakow, Poland; angelika.bieniek@student.urk.edu.pl

**Keywords:** wheat bread, arabinoxylans, molar mass, postponed baking

## Abstract

Bread is a basic element of the human diet. To counteract the process of its going stale, semi-finished bakery products are subjected to cooling or freezing. This process is called postponed baking. The aim of this work was to investigate the effect of the molar mass of rye arabinoxylans (AXs) on the properties of wheat breads baked using the postponed baking method. Breads were produced using the postponed baking method from wheat flour without and with 1 or 2% share of rye AXs clearly differing in molar masses—non-modified or modified AXs by means of partial hydrolysis and cross-linking. The molar mass of non-modified AXs was 413,800 g/mol, that of AXs after partial hydrolysis was 192,320 g/mol, and that of AXs after cross-linking was 535,630 g/mol. The findings showed that the addition of all AX preparations significantly increased the water absorption of the baking mixture, and the increase was proportional to the molar mass of AXs used as well as the share of AX preparation. Moreover, for the first time, it was shown that 1% share of partly hydrolyzed AXs, of a low molar mass, in the baking mixture had the highest effect on increasing the volume of bread and reducing the hardness of the bread crumb of bread baked using postponed baking method. It was also shown that the AXs had a low and inconclusive effect on the baking loss and moisture content of the bread crumb.

## 1. Introduction

To prevent the staling process, semi-finished bakery products are subjected to cooling or freezing [1,2,3]. The application of these techniques makes it possible to obtain products with properties similar to those produced using traditional methods, such as the preferred volume, the moisture content of the crumb, and a crispy crust. In addition, the use of these techniques ensures the availability of fresh bread and allows consumers to bake their own bakery products at home [2,3,4]. Technologies that use refrigeration or freezing are called postponed baking methods and can be divided into two groups. The first uses the freezing process. In this group, the following subgroups can be distinguished: freezing pieces of dough that have not undergone proofing, freezing pieces that have undergone proofing, and freezing pre-baked pieces. The second group consists of stopping the fermentation process at low plus temperatures, close to 0 °C, and slowing the final fermentation at 4–18 °C [3,5,6,7]. 

The volume of bread (BV) is usually determined by considering the volume of bread from the same amount of dough. The second way to measure bread volume can be determined by the specific bread volume (SBV), which means the volume of bread obtained from 100 g of flour, which also includes the dough yield. According to the literature, bread baked using the postponed baking method had a lower volume (BV) compared to bread baked using the traditional method [1]. Wheat bread baked using the postponed baking method had a similar specific volume, compared to bread baked using the traditional method [8]. However, it has been observed that thermal shock in postponed baking can reduce the volume of the final product [5,9]. The effect of carrageenan addition to bread baked using the postponed baking method on specific volume was inconclusive. Depending on the duration of storage in the frozen condition, an increase or decrease in this parameter was noted, compared to bread baked using the same method without the addition of hydrocolloids [10]. The effect of hydroxypropylmethylcellulose (HPMC) on the specific volume of wheat breads baked using the postponed baking method was also inconclusive, with reports of no influence [6] or an increase in SVB [10], compared to breads without the addition of hydrocolloids. Breads with the addition of locust bean gum, as well as a synthetic polymer (polydextrose), baked using the postponed baking method did not differ significantly in terms of specific volume from breads baked using the postponed baking method without the addition of hydrocolloids [6]. Negative effects of hydrocolloids such as inulin and oat fiber on the SBV of wheat breads have also been reported [5,6]. The share of inulin or oat fiber in the bread may have disrupted the crumb structure due to the physical disruption of the gluten mesh, which promoted crumb collapse during low-temperature storage [5,6].

The moisture content of the crumb and crust of the bread was determined separately to assess its physical properties, as well as to check water migration during baking. The effect of the postponed baking method on the moisture content of the crumb and crust of wheat bread was inconclusive [1,11].

It was shown that the addition of hydrocolloids (HPMC, pectin, oat fiber, or inulin) to wheat bread baked using the postponed baking method increased crumb moisture in the range of 2–12% [5,6,10]. However, the use of HPMC and locust bean gum in bread baked using the postponed baking method resulted in a decrease in crumb moisture in the range of 5–10% [6]. The addition of HPMC and carrageenan affected the moisture preservation of the crumb of wheat bread baked using the postponed baking method during storage, compared to bread baked using the same method without the addition of hydrocolloid [10].

One of the crucial parameters by which the quality of bread can be determined is crumb hardness. According to the literature, the effect of the postponed baking method on the hardness of the crumb of wheat bread was inconclusive [1].

The bread with the addition of HPMC baked using the postponed baking method was shown to have a lower crumb hardness, compared to bread without the addition of hydrocolloids [10,12]. HPMC has an ability to bind water, so perhaps this property inhibits the recrystallization of amylopectin, one of the factors affecting crumb hardness. The storage time of the pre-baked bread in the frozen state had no effect on the crumb hardness of the bread containing HPMC [12]. Bread with the addition of carrageenan was characterized by higher crumb hardness but only up to 14 days of frozen storage. After this time, there was no further increase in this parameter, compared to bread baked using the same method without the addition of hydrocolloids [10].

Physicochemical changes during bread storage lead to an increase in crumb hardness, a change in flavor, and a loss of crust crunchiness, and this process is called bread staling [13]. Baking bread using the postponed baking method was shown to have an effect on reducing its staling, compared to bread baked using the traditional method [1].

Arabinoxylans (AXs) belong to a group of non-starch polysaccharides that are part of the dietary fiber of cereal grains and seeds. AXs are built from a backbone composed of xylose residues, which can be substituted with arabinose residues and phenolic acid molecules, particularly ferulic acid. The content of AXs in wheat grain is 0.8–9% [14], while in rye grain it is 8–12% [15,16]. The molar mass of natural AXs from wheat grain is in the range of 176,000–381,000 g/mol [17,18,19], while from rye grain it is 197,800–2,000,000 g/mol [15,16,19,20]. The molar mass of AXs may be significantly changed by the modification. It was shown that partial enzymatic hydrolysis causes decrease and oxidative cross linking causes an increase in the molar mass of AXs [16,20]. Previous studies have shown that AXs can significantly affect the bread baking process and shape its quality parameters, with the amount of added preparation and the molar mass of AXs determining their crucial role [21].

In previous publications, there is a lack of information on the effect of AXs and their structure changed by the possible modifications on the properties of wheat bread prepared using the postponed baking method. The purpose of this study was to investigate the effect of the molar mass of rye arabinoxylans on the properties of wheat breads baked using the postponed baking method.

## 2. Results and Discussion

The composition and properties of the flour were typical of wheat flour type 750 (Table 1). The ash content of the wheat flour tested was in the range of 0.70–0.78%. The determined starch content of the flour tested was typical, as according to other studies, the content of this component in flours with similar ash content averages 70–86% [22,23]. The protein content of the tested wheat flour was also typical compared to other studies, according to which this variation ranges from 10% to 18.4% for flours with analogous ash content [22,23,24,25]. The fat content (Table 1) of the flour tested was also typical [22,24]. The content of total dietary fiber (Table 1) in wheat flour was similar to other studies, according to which this component amounts to 3.0–3.6% for flours with similar ash content [23,26]. In wheat flour, the majority of dietary fiber is the insoluble fraction (IDF). Non-starch polysaccharides, such as arabinoxylans or β-glucans, constitute a significant amount of dietary fiber [27].

The content of AXs (Table 1) in the wheat flour studied corresponded to data presented in another study, where this component ranged from 1.65% to 3.14% for flours with similar ash content [28]. The ratio of arabinose to xylose was appropriate for wheat AXs, and in previous publications, it ranged from 0.5 to 0.6 for flours with similar ash content [29].

The content of AXs in rye grain is the highest among other cereals [15,16], and therefore rye wholemeal was used as a source for AX isolation. AX preparations were obtained from rye flour and divided into three parts. One part was unmodified (AX_NM), the second part was partially hydrolyzed (AX HYD), while the third part was modified through cross-linking (AX CR). The basic chemical composition of the unmodified and modified AX preparations is shown in Table 2.

All AX preparations were characterized by a very similar composition of sugar residues released after the acid hydrolysis of AX preparations, containing mainly xylose and arabinose (Table 2). The AX content in the preparations was approximately 75%, and the A/X ratio was approximately 0.60, which was consistent with the data in other studies, in which rye AX preparations were characterized by an AX content in the range of 68–93% and an A/X ratio in the range of 0.48–0.78 [16,30,31].

The glucose determined in the preparations (Table 2) probably came from starch and/or β-glucans, the residues of which were not removed by α-amylase during the isolation and modification of AX. The resulting galactose and mannose content of the samples may also have originated from small amounts of polysaccharides, such as arabinogalactans, mannans, or other polysaccharides occurring naturally in rye grain [16,31,32].

The protein content of AX preparations was lower than in another study, where this component ranged from 8.6% to 21.7%, meaning that the preparations were characterized by higher purity [16].

The molar mass of the preparations allowed the effectiveness of AX modification to be determined. The molar mass of the AX preparation that was not modified (AX_NM) was typical for rye arabinoxylans, as according to other studies, this parameter is in the range of 200,000–500,000 g/mol [16,31,33]. The preparation of partially hydrolyzed AX (AX_HYD) was characterized by the lowest weight average molar mass, also similar to the data presented in another study, where this parameter was 219,000 g/mol [31], which also confirms the effectiveness of the modification carried out. The highest molar mass was observed for the cross-linked preparation (AX_CR), which confirms the effectiveness of the performed modification (cross-linking), and the determined Mw is in agreement with previous reports in the literature, according to which it can be about 505,000 g/mol [31].

The postponed breads were baked using type 750 wheat flour and rye AX preparations AX_NM, AX_HYD, and AX_CR. AX preparations were used instead of some of the flour at concentrations of 1% and 2%. The breads were partially pre-baked, then stored, frozen, re-baked, and analyzed. The postponed baking of wheat bread [5,6] and rye bread [34] has already been carried out in a similar way. The results of determining the properties of the breads were presented in Table 3. 

The water binding capacity (WBC) of wheat flour without and with AX preparations was determined at the same dough consistency of 500 BU. The WBC of wheat flour without the addition of AXs (Table 3) was similar to data in another study, where wheat flour with an ash content of 0.5–0.6%, was characterized by a WBC of about 60% [35]. The share of AX_CR preparation in the amount of 2% caused the highest increase (*p* < 0.05) in the water absorption of wheat flour, which is comparable with data in previous publications, where an AX additive with a molar mass of 620,000 g/mol was used [36]. The share of AX_HYD in the amount of 1% resulted in the lowest increase (*p* < 0.05) in the WBC of wheat flour, which could be due to the lower molar mass of AXs (Table 2). Previous publications have reported that the addition of AXs with a molar mass of 70,000–201,600 g/mol had a positive effect on increasing the water absorption of wheat flour [35,37,38].

The consequence of the increased water absorption of flour and the increased addition of water to the dough in order to obtain optimal consistency is a higher dough yield (DY, Table 3), which is economically important for the baking industry, as more bread can be produced from the same amount of flour [33,39]. The dough yield (DY) without the addition of AX preparations was similar to that in the literature, where DY using flour with a similar ash content was approximately 162 g [25].

The AX_CR preparation had the highest effect on improving DY, especially at 2% share (Table 3). The application of AX_HYD at 1% share had the lowest effect on improving DY. The share of the AX_NM preparation had a positive effect on DY.

Table 3 presents the results regarding baking loss, which was determined immediately after baking (BL) and after cooling the breads (TBL). The baking loss (BL) in wheat bread without the addition of AXs was 14%, but there are no data in the literature relating to BL in baking wheat bread using the postponed baking method. The total baking loss (TBL) of wheat bread without the addition of AXs was lower than in another study, where the TBL was approximately 21% for bread baked from wheat flour with an ash content of 0.53% [40].

Breads with AX preparations had higher (*p* < 0.05) baking loss (BL) and total baking loss (TBL) compared to breads without AXs. A 1% share of the AX_CR preparation resulted in the highest baking loss and total baking loss of the bread, while a 2% share of the same preparation resulted in the lowest BL and TBL, compared to bread without added AXs. The share of AX_NM and AX_HYD affected the increases in baking loss and total baking loss of the breads, and an increase in these parameters was noted as the share of AX preparations increased. The higher baking loss of breads with AX preparations could result from their bigger volume, and therefore a bigger evaporation area [41]. TBL increased after the cooling of all breads due to further water evaporation [42].

Table 3 shows the results regarding the volume of breads baked from the same amount of dough (BV). Comparing the wheat breads obtained using the postponed baking method, the bread baked without AXs (Table 3) had higher volume compared to another study [1]. The difference may have resulted from the wheat flour used; unfortunately, the authors did not define the type in the publication [1]. 

The AX_HYD preparation had the largest effect on volume (BV), compared to bread without AXs (Table 3). The AX_NM and AX_CR preparations at 1% share did not affect the volume (BV) of the breads, while a decrease in BV was noted when a 2% share of the same preparations was used, compared to breads without the AX preparation. 

In addition to determining the volume of bread from the same amount of dough, the specific volume (SBV), which is the volume of bread obtained from 100 g of flour, was also determined. Wheat bread without AXs obtained using the postponed baking method had a similar specific volume, compared to other studies, where SBV was 3–6 cm^3^/g [5,6,25].

The addition of AXs had a positive effect on the specific volume (SBV) of wheat breads baked using the postponed baking method, excluding the share of AX_NM at 2% (Table 3). The share of AX_HYD had the largest effect (*p* < 0.05) on increasing the specific volume of the breads. According to another study, there is a tendency that the addition of hydrocolloids (carrageenan and HPMC) increases SBV in wheat bread baked using the postponed baking method [10]. The share of AX_NM in the amount of 2% had a negative impact on the specific volume of wheat bread baked using the postponed baking method, which is compatible with information in the literature on the negative effect of hydrocolloids, such as inulin or oat fiber, on the SBV of wheat breads [5,6]. The share of the AX_CR preparation had a low impact on increasing the SBV of wheat bread baked using the postponed baking method. The observed differences in SBV may be related to the type of AX preparation used in bread baking [5,6]. It is known that water-soluble AXs of a high molar mass form a gel network which can probably enhance the gluten network. The gluten network can be stabilized by diferulic acid linkages formed between AXs or between AX molecules and proteins [21,43]. However, it is worth mentioning that the adverse effect of AXs may be due to the presence of high-molar-mass, water-insoluble AX fractions that inhibit gluten network formation. This is due to the competition of water-insoluble fractions of AXs with gluten for water, and results in reduced gas retention, causing the bread to not rise well [32].

Wheat breads without AXs, baked using the postponed baking method, had similar crumb hardness compared to another study, where this parameter constituted about 7 N [1]. The share of AX preparations AX_HYD and AX_CR in wheat bread prepared using the postponed baking method reduced crumb hardness (Table 3), which is consistent with other studies, where the addition of hydrocolloids also caused a reduction in this parameter [1,12,34]. However, the share of AX_NM in the amount of 2% caused an increase in crumb hardness, compared to bread without the AX addition, which was also noted in another study where the addition of carrageenan was used [10]. Despite the lower water absorption of flour containing hydrolyzed AXs (AX_HYD), compared to AX_NM and AX_CR, hydrolyzed, soluble, short-chain AXs better stabilized gas bubbles in the dough, which resulted in bread with a larger volume and a decreased crumb hardness.

Table 4 shows the results of the bread crumb moisture. The moisture content of the bread crumb in the central and peripheral parts (close to the crust) was determined to observe water migration in the crumb of the breads. The moisture content of the crumb in the central part (Table 4) of wheat breads baked using the postponed baking method was comparable to the data in another study [1]. The moisture content of the peripheral part of the crumb of bread baked using the postponed baking method has not been studied previously.

The share of AX_CR increased (*p* < 0.05) the moisture content of the crumb in the central part compared to bread without AXs. This is consistent with previous studies in which the addition of hydrocolloids to wheat bread baked using the postponed baking method increased this parameter [5,6,10]. However, it was noted that the share of AX_HYD and AX_NM preparations in the amount of 1% resulted in a decrease in bread crumb moisture, compared to bread without the addition of AXs. According to another study, the addition of locust bean gum and HPMC caused a decrease of bread crumb moisture of wheat bread baked using the postponed baking method [6]. It was observed that the moisture contents in the central and peripheral parts of the bread crumb are very similar (Table 4). So, it may be concluded that AXs facilitated the alignment of the crumb moisture of the breads in the central and peripheral parts of the bread.

## 3. Materials and Methods

Wheat flour type 750 (PZZ Krakow, Poland, Batch number 02.2024P4DK050923) was used for baking. The material used for the isolation of arabinoxylans was flour produced using a laboratory method from the rye variety Amilo (Danko, Poland). Other baking additives included freeze-dried *Saccharomyces cerevisiae* yeast from Lesaffre, France, and salt (NaCl) from POCh, Poland.

### 3.1. Isolation and Modification of Water-Soluble Non-Modified Rye Arabinoxylans 

The water-extractable AXs were isolated and modified according to the methods of Buksa et al. [12].

#### 3.1.1. Isolation of Arabinoxylans

In short, 100 g of wholemeal rye flour was subjected to cereal enzyme inactivation with 500 mL of 80% *v/v* EtOH at 90 °C for 2 h. The ethanol solution was removed, and the precipitate was dried at 40 °C for 20 h, after which it was extracted with 2 L of water at 25 °C for 6 h. As a next step, the suspension was centrifuged. The clear supernatant was boiled to coagulate soluble proteins, and then cooled and incubated with α-amylase at 37 °C for 2 h. The solution was boiled, and then 20 g/L cellite was added and filtered using a Buchner flask. The clear filtrate was poured into a 4-volume solution of ethanol and acetone (1:1). The sediment was centrifuged, and then frozen at −18 °C and stored for further processing (modifications), or washed twice with ethanol/acetone and twice with acetone only. After the last centrifugation, the precipitate of AXs was dried at 50 °C for 2 h. The yield of the non-modified AX preparation (denoted as AX_NM) was 2.8 g/100 g of the wholemeal.

#### 3.1.2. Modification of Isolated Rye Arabinoxylans Using Cross-Linking

A total of 15 g of frozen precipitate was thawed and dissolved in 40 mL of deionized water with intense stirring at 50 °C for 6 h. The solution was then cooled to 25 °C, and hydrogen peroxide (1 μg/g AXs) and peroxidase (5 U/g AXs) were added and reacted for 15 min. The process was stopped by flooding the solution with a 4-volume solution of ethanol and acetone (1:1). The AX precipitate was centrifuged and washed twice with ethanol/acetone and twice with acetone only to remove all water from the sample. After the last centrifugation, the precipitate of AXs was dried at 50 °C for 2 h. The resulting preparation of cross-linked AX was denoted as AX_CR.

#### 3.1.3. Modification of Isolated Rye Arabinoxylans Ssing Partial Enzymatic Hydrolysis

A total of 15 g of frozen precipitate was thawed and dissolved in 40 mL of deionized water with intense stirring at 50 °C for 6 h. After cooling the solution to 30 °C, xylanase (endo-β-1,4-xylanase) of *Thermomyces lanuginosus* (Merck Life Science Sp.z.o.o., an affiliate of Merck KGaA, Darmstadt, Germany) was added at 375 FXU/g AX and incubated at 37 °C for 30 min. After boiling, the sample was centrifuged, and the resulting supernatant was transferred to a 4-fold volume of ethanol/acetone solution (1:1). The precipitated AXs were centrifuged and washed twice with ethanol/acetone and twice with acetone only. After the last centrifugation, the precipitate of AXs was dried at 50 °C for 2 h. The resulting preparation of partly hydrolyzed AX was denoted as AX_HYD.

### 3.2. Determination of the Monosaccharide Composition of Arabinoxylans

The monosaccharide composition of AX preparations was determined using HPLC/RI method after acid hydrolysis according to Buksa et al. [12]. In short, 20 µL of 4 mg/mL hydrolyzed sample solution was injected into an HPLC system (Knauer Wissenschaftliche Geräte GmbH, Berlin, Germany).

The estimated total sugar content was calculated as the sum of the contents of all sugar residues after acid hydrolysis. The estimated content of AXs in the preparations was calculated by taking the sum of the content of arabinose and xylose after acid hydrolysis and multiplying the result by a factor of 0.88, which is calculated from the molecular weight of AX monomer/molecular weight of pentose (132/150 = 0.88). The ratio of arabinose to xylose (A/X) was calculated by dividing the content of arabinose by the content of xylose after acid hydrolysis. The estimated glucan content was calculated by multiplying the glucose content (Glc) by a factor of 0.9 (Table 2), which is calculated from the molecular weight of starch monomer/molecular weight of glucose (162/180 = 0.9).

### 3.3. Determination of Molecular Properties of Arabinoxylans

The distribution of molar mass of AX was evaluated using HPSEC/RI according to Buksa et al., 2016 [20]. The chromatographic system consisted of a Knauer chromatograph (Knauer, Germany), equipped with a combination of OHpak SB-806HQ and SB-804HQ (Shodex, Japan) columns and a refractometric detector (Knauer, Germany). A total of 100 mM NaNO_3_ was used as eluent at a flow rate 0.6 mL/min. The separation was performed at a column temperature of 60 °C. The calibration of the HPSEC system was performed with pullulan standards (Shodex Standard, Macherey—Nagel) with known molar masses (P-5, 10, 100, 400, and 800) and arabinose (Sigma-Aldrich). The molar mass distribution and apparent average molar mass *Mw* (related to pullulan standards) were calculated using the software programs Eurochrom (Knauer) and Clarity (ver. 4.0.1.700, DataApex).

### 3.4. Baking Bread Using the Postponed Baking Method and Determination of Bread Parameters

Doughs were made from type 750 wheat flour and rye AXs. The breads were baked according to Buksa et al. [34]. According to the base recipe, the dough for one wheat bread was made from 100 g of wheat flour, water in the amount determined by the farinograph (dough with a consistency of 500 BU, determined according to ICC-Standard No. 115/1), 1.8% salt, and 3.5% yeast (Supplementary Table S1). The mentioned recipe was used to make the dough and bread, constituting the control sample. In making the other doughs, AX preparations of 1% and 2% by weight of flour were added in place of flour. In the farinograph mixer, doughs of equal consistency of 500 BU were made from the tested flours, from which, after shaping the pieces (60 g) and fermentation (60 min), the breads were baked in aluminum molds, in a Viva-Meteor oven (Victus Srl, Costa di Rovigo, Italy). The molds with the doughs were placed in an oven preheated to 160 °C; the temperature was gradually increased to reach 190 °C and kept at 190 °C for 3 min, without allowing the crust to color. The partly baked breads were then cooled to room temperature and frozen in a blast freezer at −23 °C until a temperature of −23 °C was reached inside the loaves. The frozen breads were stored in the freezer for 2 weeks. After defrosting at 60 min, the breads were re-baked at 230 °C for 17 min.

#### 3.4.1. Volume

After cooling, the volume of the baked breads was determined by the 3-dimensional laser-based scanner Volscan Profiler (Stable Microsystems, Godalming, UK), according to the manufacturer’s manual.

#### 3.4.2. Moisture

The moisture content of the crumb was determined by drying for 1 h at 130 °C, according to AOAC 925.10 (2006).

To determine the moisture content of the crumb, samples were taken from the central part of the bread and from the peripheral part, which was very close to the crust. 

#### 3.4.3. Texture

Texture parameters were determined using the texture analyzer TA.XT Plus (Stable Microsystems, GB) according to the standard program, at the compression rate of 5 mm/s. A sample of bread crumb, taken from the base of the loaf, with a height of 30 mm, was pressed to reach 10 mm maximum strain using a P/20 aluminum compression plate with a diameter 15 mm, in two cycles with a 5 s delay. From the resulting parameters of TPA (Texture Profile Analysis), only the hardness of the crumb was used as an indicator of textural properties. The calculations were performed using the attached software Texture Exponent (Stable Microsystems, Godalming, UK).

#### 3.4.4. Statistical Analysis

All experiments or tests were performed at least in triplicate. A statistical analysis of variance (ANOVA) was performed in order to determine statistical significance of the observed differences among mean values (Tukey’s test at a significance level of 0.05). The statistical analysis was performed using Statistica v. 9.0 software (StatSoft, Inc., Tulsa, OK, USA).

## 4. Conclusions

The non-modified (AX_NM) rye AXs obtained through the laboratory method have an average molar mass of 412,000 g/mol. After modifications, the highest molar mass was observed for the AXs in the AX_CR preparation, while the lowest for the AXs in the AX_HYD preparation. 

The share of AX preparations significantly increased the water absorption of baking mixtures and the yield of dough, proportionally to the molar mass of the AXs used and their share in the baking mixtures. The most effective was cross-linked AXs (AX_CR) at 2% share in the flour that cause an approx. 9% increase in the dough yield.

The addition of AX preparations significantly improved the quality of breads baked using postponed baking method. Partly hydrolyzed AXs (LP_HYD), of low molar mass and high solubility in water, especially at 1% share in the flour, had the highest effect on increasing the bread volume (by approx. 7%) and reducing the hardness of the bread crumb (by approx. 20%). The arabinoxylans had a low and inconclusive effect on the baking loss and the moisture content of the bread crumb of breads baked using postponed baking method.

AX preparations, especially those containing partly hydrolyzed AXs, may be recommended as a bread improver for bread baked using postponed baking methods.

## Figures and Tables

**Table 1:** Chemical composition of flour.

Component	Wheat Flour Type 750
Starch [%]	72.2 ± 1.1
Protein [%]	12.6 ± 0.4
Fat [%]	1.8 ± 0.0
Total dietary fiber—TDF [%]	3.6 ± 0.3
Soluble dietary fiber—SDF [%]	1.7 ± 0.1
Insoluble dietary fiber—IDF [%]	1.9 ± 0.1
Arabinoxylans [%]	2.8 ± 0.1
Arabinose/Xylose ratio	0.57 ± 0.03
Ash [%]	0.73 ± 0.01

**Table 2:** Chemical composition and properties of water-soluble arabinoxylans (AX) obtained from rye wholemeal.

Component	AX_NM *	AX_HYD *	AX_CR *
Glucose [%]	9.9 ± 0.4	9.4 ± 0.5	9.5 ± 0.2
Xylose [%]	53.7 ± 0.5	52.6 ± 0.5	54.1 ± 1.3
Galactose [%]	2.2 ± 0.4	2.2 ± 0.6	2.1 ± 0.4
Arabinose [%]	32.6 ± 0.6	32.6 ± 0.5	31.9 ± 1.1
Mannose [%]	0.6 ± 0.1	0.7 ± 0.1	0.7 ± 0.1
AX [%]	76.0 ± 1.0	75.0 ± 0.8	75.7 ± 0.2
Arabinose/Xylose ratio	0.61 ± 0.01	0.62 ± 0.01	0.59 ± 0.03
Glucan [%]	8.9 ± 0.3	8.4 ± 0.5	8.5 ± 0.2
Total sugar content [%]	99.0 ± 1.7	97.5 ± 1.3	98.3 ± 0.1
Protein [%]	7.2 ± 0.5	7.4 ± 0.6	7.5 ± 0.7
Molecular parameters of AX			
M_w_ [g/mol] **	413,800	192,320	535,630
M_n_ [g/mol] **	6980	4560	8460
Ð **	59.3	42.2	63.3

* AX_NM—non-modified AX; AX_HYD—hydrolyzed AX; AX_CR—cross-linked AX; ** M_w_/M_n_—weight/number average molar mass; Ð—dispersity.

**Table 3:** Properties of wheat bread in the postponed baking method without (control) or with a 1% or 2% share of AX preparations.

Parameters **	Control *	AX_NM *		AX_HYD *		AX_CR *	
	0%	1%	2%	1%	2%	1%	2%
WBC [%]	57.6 ± 0.3 ^a^	61.0 ± 0.2 ^c^	65.1 ± 0.1 ^f^	60.2 ± 0.4 ^b^	62.1 ± 0.2 ^d^	63.4 ± 0.1 ^e^	66.4 ± 0.2 ^g^
DY [%]	157.6 ^a^	161.0 ^c^	165.1 ^f^	160.2 ^b^	162.1 ^d^	163.4 ^e^	166.4 ^g^
BL [%]	14.0 ± 0.3 ^a^	14.2 ± 0.0 ^a^	14.9 ± 0.6 ^a^	14.4 ± 0.2 ^a^	14.8 ± 0.5 ^ab^	15.8 ± 0.7 ^b^	14.6 ± 0.5 ^a^
TBL [%]	15.6 ± 0.3 ^ab^	15.8 ± 0.0 ^ab^	16.4 ± 0.6 ^bc^	15.9 ± 0.3 ^ab^	16.3 ± 0.0 ^b^	17.1 ± 0.5 ^c^	15.8 ± 0.5 ^ab^
BV [cm^3^]	151.5 ± 1.5 ^c^	151.0 ± 1.0 ^c^	137.0 ± 0.0 ^a^	162.0 ± 1.0 ^e^	153.5 ± 1.5 ^d^	150.0 ± 0.0 ^c^	146.5 ± 0.5 ^b^
SBV (cm^3^/100 g of Flour)	397.9 ± 3.9 ^b^	405.2 ± 2.7 ^c^	376.8 ± 2.8 ^a^	432.0 ± 2.7 ^g^	414.5 ± 4.1 ^f^	408.5 ± 0.0 ^e^	406.3 ± 1.4 ^cd^
Crumb Hardness (N)	7.6 ± 0.3 ^c^	6.5 ± 0.2 ^b^	8.6 ± 0.3 ^d^	6.0 ± 0.2 ^a^	5.9 ± 0.3 ^a^	6.8 ± 0.1 ^b^	7.2 ± 0.1 ^c^

* Control—wheat bread without AXs; AX_NM—wheat bread with non-modified AXs; AX_HYD—wheat bread with hydrolyzed AXs; AX_CR—wheat bread with cross-linked AXs. ** WBC—water binding capacity, DY—dough yield, BL—baking loss of hot bread, TBL—total baking loss after cooling; BV-Bread volume; SBV—Specific bread volume. Mean values marked with the same letters in particular rows are not statistically significantly different at *p* < 0.05.

**Table 4:** Moisture content of the crumb in the central part and in the peripheral part of wheat bread in the postponed baking method without (control) or with a 1% or 2% share of AX preparations.

AX Preparation *	Crumb Moisture—Central Part (%)			Crumb Moisture—Peripheral Part (%)		
	0%	1%	2%	0%	1%	2%
Control	43.7 ± 0.03 ^c^			43.6 ± 0.1 ^bc^		
AX_NM		42.9 ± 0.6 ^ab^	44.3 ± 0.4 ^cd^		42.5 ± 0.1 ^a^	43.8 ± 0.2 ^bc^
AX_HYD		42.3 ± 0.2 ^a^	43.5 ± 0.4 ^bc^		42.4 ± 1.0 ^ab^	43.2 ± 0.7 ^abc^
AX_CR		43.2 ± 0.8 ^b^	45.03 ± 0.2 ^e^		43.4 ± 0.5 ^bc^	45.6 ± 0.1 ^d^

* Abbreviations of sample names as in Table 3, Mean values marked with the same letters are not statistically significantly different at *p* < 0.05.

## Data Availability

Data are contained within the article and supplementary materials.

## Supplementary Materials

The following supporting information can be downloaded at: https://www.mdpi.com/article/10.3390/molecules29040904/s1, Table S1. The amounts of ingredients used for dough preparation from 100 g of flour.
