# Supplementary material for: The Influence of Arabinoxylans on the Properties of Wheat Bread Baked Using the Postponed Baking Method
Source: Molecules. 2024 Feb 18;29(4):904. doi: 10.3390/molecules29040904 (PMC10893419; doi:10.3390/molecules29040904)
Supplement: Supplementary file 1 [file molecules-29-00904-s001.zip › molecules-2849470-supplementary.pdf]

Supplementary material

Table S1. The amounts of ingredients used for dough preparation from 100 g of flour.

AX preparation*	Share of AX [%]	Share of AX [g]	Share of flour [g]	Share of yeast [g]	Share of salt [g]
Control	0	0	100	3.5	1.8
AX_NM	1	1	99	3.5	1.8
AX_NM	2	2	98	3.5	1.8
AX_HYD	1	1	99	3.5	1.8
AX_HYD	2	2	98	3.5	1.8
AX_CR	1	1	99	3.5	1.8
AX_CR	2	2	98	3.5	1.8

*Control- wheat bread without AX; AX_NM- wheat bread with non-modified AX; AX_HYD- wheat bread with hydrolyzed AX; AX_CR-wheat bread with cross-linked AX.
